# Effect of Preheating Conditions on the Mechanical Reliability of HDPE Extrusion-Welded Structures for Medical Devices

**DOI:** 10.3390/polym18121467

**Published:** 2026-06-11

**Authors:** Chung-Woo Lee, Eunho Choe

**Affiliations:** EM Actuators Lab, Korea Institute of Medical Microrobotics, Gwangju 61011, Republic of Korea; lcw@kimiro.re.kr

**Keywords:** high-density polyethylene (HDPE), extrusion welding, preheating conditions, mechanical properties, medical device manufacturing

## Abstract

Polymers are increasingly used as structural materials in medical devices due to their lightweight, chemical resistance, and electrical insulation properties. High-density polyethylene (HDPE) is widely applied; however, the fabrication of large or thick components often requires extrusion welding. Since joint performance directly affects structural reliability, controlling welding quality is essential. In this study, the effects of preheating conditions on the mechanical performance of HDPE extrusion-welded joints were systematically investigated. Preheating temperature and hot-air movement speed were selected as key variables, and their influence on tensile, flexural, and elongation properties was evaluated experimentally. The results provide insight into the role of preheating in weld quality and offer guidance for process optimization in medical device applications.

## 1. Introduction

In recent years, the medical device industry has increasingly adopted polymers as structural materials in place of conventional metals [1,2,3,4,5]. This trend is driven by the demand for lightweight design, enhanced chemical resistance, improved electrical insulation, and greater design flexibility. In particular, medical electrical equipment and diagnostic or therapeutic devices are frequently exposed to repeated cleaning and disinfection processes while being required to maintain mechanical stability and electrical safety. Consequently, the selection and processing of polymer-based structural materials play a critical role in ensuring device reliability and patient safety [6,7,8,9,10].

High-density polyethylene (HDPE) is a polymer characterized by excellent chemical resistance, low moisture absorption, high impact strength, and superior electrical insulation properties [11,12,13]. HDPE is commonly employed in medical equipment housings, fluid storage containers, protective covers, cable guides, and mechanically supportive structural components. In addition to its functional advantages, HDPE offers cost-effectiveness and ease of processing, making it attractive for large-scale medical device manufacturing. However, HDPE components are often required in the form of thick plates or large structural parts, which are difficult to fabricate as monolithic components using conventional molding techniques [14,15,16,17,18].

Extrusion welding, which joins base materials using molten filler material, is widely adopted as a practical and reliable method for joining thick polymer components due to its high joint strength and relatively simple process control. In particular, joining processes are essential in the fabrication of large-scale medical equipment structures, where the mechanical performance of the joints directly determines the overall structural stability. Therefore, ensuring the quality of the extrusion welding process is of critical importance [19,20,21,22,23,24,25,26,27].

The mechanical performance of HDPE extrusion-welded joints is influenced by various process parameters, among which preheating conditions play a crucial role. Preheating the base material prior to welding promotes molecular interdiffusion across the weld interface, reduces thermal gradients, and affects crystallization behavior during cooling. Proper preheating can enhance joint strength and reduce interfacial defects, whereas insufficient or excessive preheating may lead to poor bonding or thermal degradation [28,29,30,31,32,33,34].

Despite its importance, previous studies on HDPE extrusion welding have primarily focused on parameters such as extrusion temperature, welding speed, bead geometry, and tool design. In contrast, the effects of preheating conditions—particularly preheating temperature and hot-air movement speed—have not been sufficiently quantified, especially for thick-section HDPE components. This lack of systematic understanding represents a significant knowledge gap, particularly in medical device manufacturing, where validated process windows and documented process control are essential for regulatory compliance.

In this study, the effects of preheating conditions on the mechanical performance of HDPE extrusion-welded joints are systematically investigated through experimental analysis. Preheating temperature and hot-air movement speed are selected as key process variables, and their influence on tensile, flexural, and elongation properties of the welded joints is evaluated. This study was conducted on structural HDPE components used in large medical equipment as a preliminary investigation prior to studies involving implantable medical devices. Representative applications include equipment housings, insulation covers, cable management structures, fluid storage tanks, and supporting frames used in large medical systems, where reliable welded joints are essential for structural integrity and long-term durability. The findings of this study provide quantitative insights into the role of preheating in HDPE extrusion welding and offer practical guidance for the design and process validation of welded HDPE structures in medical devices, where mechanical reliability and manufacturing reproducibility are of paramount importance.

## 2. Materials and Methods

### 2.1. Materials and Equipment

For the experimental investigation, HDPE base plates with dimensions of 115 mm (length) × 250 mm (width) × 12 mm (thickness) were prepared, each featuring a precisely machined V-groove with a bevel angle of 30° to ensure consistent penetration and fusion during the extrusion welding process. The base material used was high-density polyethylene (EcoMarine grade, Busan, Republic of Korea). The filler material was a black HDPE wire with a nominal diameter of 4 mm (Huiyang, Busan, Republic of Korea), chosen to match the base material in chemical composition and thermal behavior to ensure interfacial compatibility and minimize defects such as voids or incomplete fusion. The material properties listed in Table 1 were obtained from a combination of experimental measurements and manufacturer data. Specifically, the tensile strength, bending strength, and melt index of the HDPE base material were experimentally determined prior to welding, whereas the density, elongation, and melting point of both the base plate and filler wire were referenced from the manufacturer’s mill sheets to ensure material consistency and compatibility. These properties were used as a baseline for evaluating the effects of processing parameters on joint performance.

For the welding process, an extrusion welding device (Extruder; Sinwoo, Republic of Korea, D4), capable of wire feeding, was mounted on a six-axis articulated robot (Yaskawa, Japan, MH6) to control the process parameters. The welding torch was positioned at a 90° working angle to provide consistent pressure to the weld joint. A hot-air gun (Leister, Kaegiswil, Switzerland) was attached in front of the extruder to preheat the base material.

**Figure 1:**
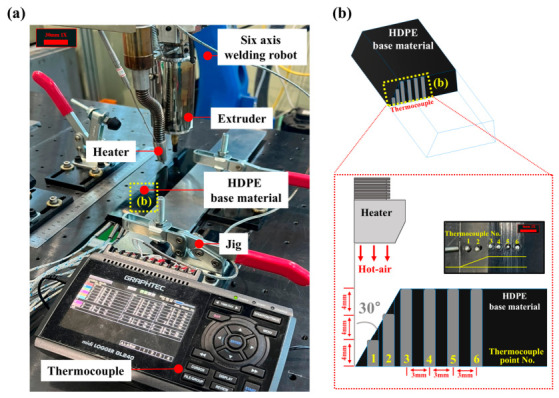
Experimental setup: (**a**) welding equipment configuration; (**b**) thermocouple installation positions.

To evaluate the influence of preheating temperature on weld performance, six thermocouples were installed to measure the surface temperature of the base material, and a data logger (Graphtec, Yokohama, Japan) was used to record the data. Figure 1 illustrates the experimental setup and the thermocouple positions used in this study. To improve the repeatability and reliability of the experiments, a clamp-type jig was customized. Temperature data were recorded at a sampling rate of 4 frames per second using the thermocouples. Figure 1b illustrates the positions of the thermocouples according to the weld geometry. The weld was fabricated in a V-groove configuration, and three thermocouples were inserted into holes that penetrated from the bottom to the surface along the slope of one side. Additionally, four thermocouples were placed on the weld surface at 3 mm intervals to measure the temperature.

**Figure 2:**
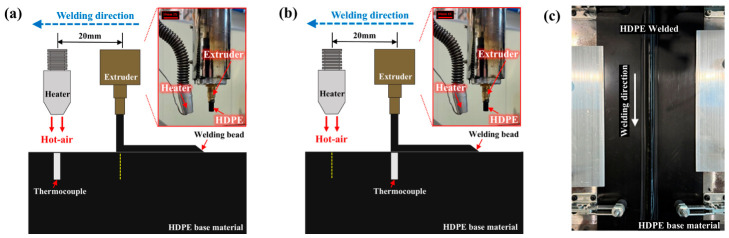
Experimental setup for HDPE extrusion welding: (**a**) hot-air preheating system; (**b**) extrusion welding configuration; (**c**) welded specimen.

**Figure 3:**
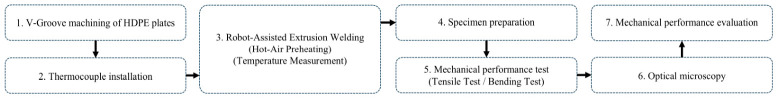
HDPE welding and results analysis process.

Figure 2 schematically illustrates the moment when the hot-air gun and the wire extrusion nozzle are aligned with the thermocouples attached to the HDPE base material. Since the hot-air gun is positioned ahead of the extruder in the welding direction, the extruder passes over the weld zone after the hot-air gun. The preheating temperature at the actual weld interface is defined in this study as the temperature measured by the thermocouple when the extruder nozzle passes over it. In addition, Figure 2c shows the actual welded specimen, clearly indicating the weld bead morphology and the alignment along the welding direction. Figure 3 shows the HDPE welding and results analysis process.

### 2.2. Experimental Design

Experiments were conducted on a base material with a 30° V-groove to determine the optimal process parameters for butt welding with a 1 mm root gap. The extrusion discharge rate was determined by the rotational speed of the extruder screw, which directly controls the melt flow rate, and was experimentally verified by measuring the amount of material extruded over one minute under steady-state conditions. Based on the measured discharge rate, the corresponding welding speed was calculated by comparing the volume of extruded material with the weld joint volume to ensure complete bead filling in accordance with DVS guidelines. Consequently, the experimental ranges of extrusion rate (0.2–0.7 g/s) and hot-air movement speed (10–35 cm/min) were selected to produce uniform and defect-free weld beads. During welding, the extruder barrel temperature was maintained at 240 °C to ensure sufficient melt flow, and the hot-air preheating temperature was held constant at 260 °C to promote proper fusion at the interface. Each condition was repeated three times to verify process stability and consistency of bead geometry. This experimental setup provides a rational and reproducible basis for correlating welding parameters, preheating conditions, and the resulting weld quality. Table 2 shows the HDPE welding conditions.

### 2.3. Evaluation Method

**Figure 4:**
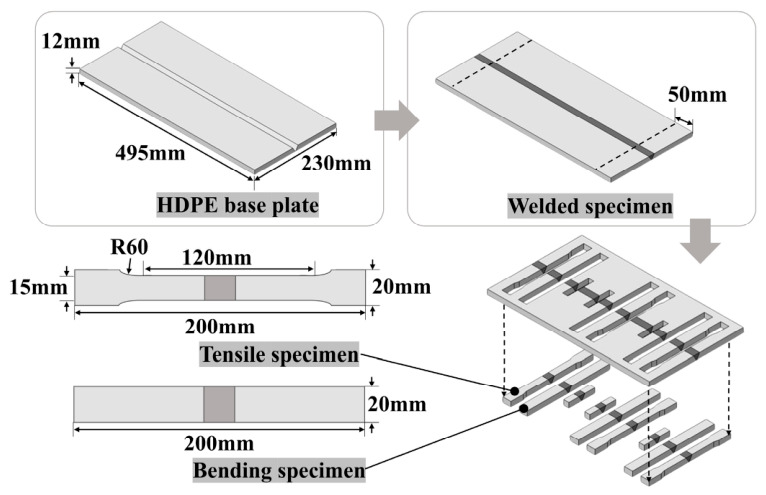
Preparation of mechanical property test specimens.

The mechanical performance of the HDPE welded joints was evaluated through tensile and bending tests. For the preparation of test specimens, approximately 50 mm was cut from both ends of each welded plate to exclude regions near the weld initiation and termination points, where material feeding tends to be unstable and weld imperfections may occur. Therefore, specimens were extracted from the central region of the weld to ensure consistent material supply and stable weld quality. A schematic illustration of the specimen preparation process is shown in Figure 4.

Two types of tensile specimens were fabricated: (i) specimens with the weld bead in-tact and (ii) specimens with the weld bead removed. Although specimens retaining the bead exhibit non-uniform cross-sections, making quantitative comparison difficult, they are useful for analyzing fracture location and fracture surface morphology. Tensile testing was performed using an universal testing machine(Instron, Norwood, MA, USA) at a crosshead speed of 50 mm/min. Each condition was tested three times, and the average value was reported.

Unlike ASTM or ISO standards commonly applied to polymer materials, the EN standard provides explicit evaluation criteria for thermoplastic welded joints and accommodates a wider applicable thickness range, making it particularly suitable for HDPE weld assessment.

Bending tests were performed using the same testing equipment (Instron 34SC-5) as the tensile test, with three specimens tested per condition at a crosshead speed of 50 mm/min. After testing, the fracture location within the weld was examined to assess weld quality. For microstructural observation, cross-sectional specimens were extracted without removing the bead and examined using optical microscopy (OM). The weld cross-sections were evaluated following the DVS 2202-1 standard to identify possible defects generated during the welding process, such as voids, lack of fusion, or inclusions.

## 3. Experimental Results

### 3.1. Welding Results

**Figure 5:**
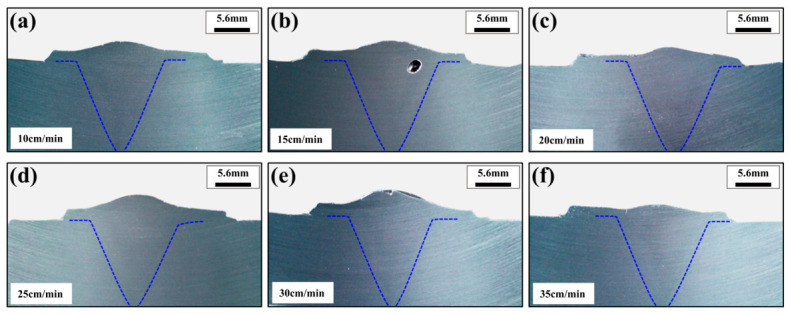
Cross-sectional morphologies of welded specimens fabricated under different extrusion rates and travel speeds: (**a**) 0.2 g/s, 10 cm/min; (**b**) 0.3 g/s, 15 cm/min; (**c**) 0.4 g/s, 20 cm/min; (**d**) 0.5 g/s, 25 cm/min; (**e**) 0.6 g/s, 30 cm/min; (**f**) 0.7 g/s, 35 cm/min.

All six welding experiments resulted in good welds. Figure 5 shows the cross-sectional results of the welds according to the hot-air movement speed. Table 3 presents the measured bead geometries of the welded joints with respect to the hot-air movement speed. Because the bead area filled under each combination of discharge and hot-air movement speed was calculated, no significant differences in bead geometry were observed.

The evaluation of HDPE weld bead geometry in this study was performed in accordance with DVS 2202-1, which provides visual inspection criteria for classifying and assessing defects in thermoplastic welded joints. This guideline specifies standards for bead shape, voids, undercuts, and notches, offering quantitative limits particularly for bead height. According to DVS 2202-1, the acceptable range for bead height is defined as 0.1 s ≤ Height ≤ 0.3 s, where s is the base material thickness. For the HDPE plates used in this experiment (s = 12 mm), the corresponding criterion requires the bead height to fall within 1.2 mm ≤ Height ≤ 3.6 mm. Although DVS 2202-1 does not impose strict dimensional limits on bead width, it emphasizes that the bead surface should be smooth and free from notches or irregularities.

Measurements of the cross-sectional dimensions confirmed that all weld beads satisfied the bead shape and height requirements specified by the DVS 2202-1 standard, indicating that the weld quality was within the acceptable range for thermoplastic joints.

### 3.2. Preheating Results over Time

**Figure 6:**
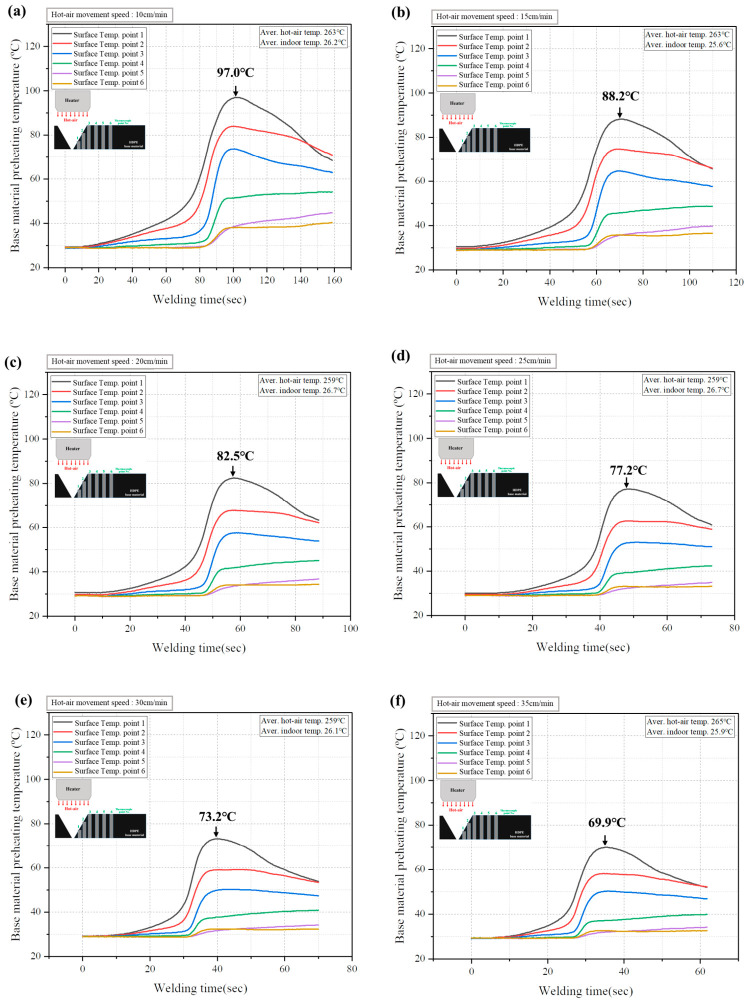
Temporal variation in preheating temperature at each thermocouple position under different hot-air movement speeds: (**a**) 10 cm/min, (**b**) 15 cm/min, (**c**) 20 cm/min, (**d**) 25 cm/min, (**e**) 30 cm/min, (**f**) 35 cm/min.

Figure 6 shows the changes in preheating temperature at each thermocouple point over time. As indicated in Figure 6, due to the offset distance between the hot-air heater and the wire extrusion nozzle, the time required to reach the maximum temperature differed depending on the thermocouple’s position. At thermocouple points 1–3, which directly received the preheating hot air, the maximum temperature was reached before the welding moment, whereas at points 4–6, which were not directly exposed to the hot air, the maximum temperature was reached later over time. This suggests that the temperature rise at thermocouple points 4–6 was caused by heat conduction through the HDPE base material rather than direct hot-air exposure.

### 3.3. Preheating Results by Location

**Figure 7:**
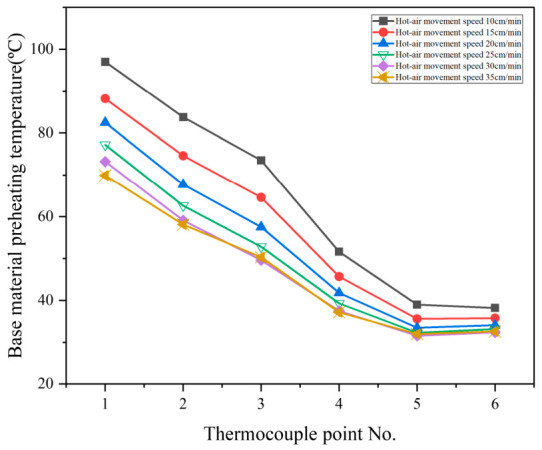
Preheating temperature at the welding moment under different hot-air movement speeds.

Figure 7 shows the preheating temperature at the welding moment measured at different locations within the base material. Regardless of the hot-air movement speed, the preheating temperature decreased as the thermocouple was positioned farther from the center of the base material. This is considered to result from the localized application of the hot-air preheating on the weld area. However, as indicated by the results in Figure 6, despite the V-groove geometry, thermocouple point 3—which is physically closer to the hot-air nozzle than thermocouple points 1 and 2 in terms of vertical distance—did not reach a higher temperature. This suggests that the lateral distance from the center of the hot-air stream has a greater effect on preheating temperature than vertical height relative to the hot-air nozzle.

**Figure 8:**
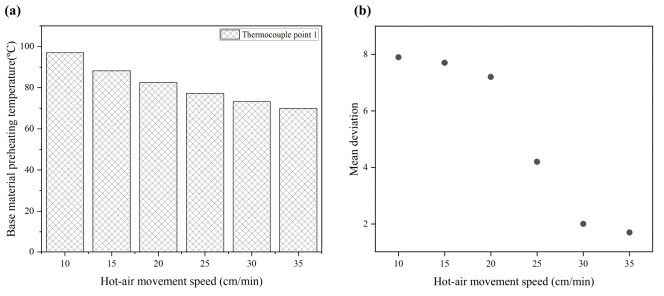
Maximum temperature at thermocouple point 1: (**a**) maximum temperature at point 1 under different hot-air movement speeds; (**b**) mean deviation of temperature at point 1 under different hot-air movement speeds.

Figure 8a shows the maximum preheating temperature measured at thermocouple point 1, which is closest to the center of the hot-air stream, while Figure 8b shows the mean deviation of preheating temperature according to hot-air movement speed. As observed in Figure 8a, the preheating temperature of the base material decreased linearly as the hot-air movement speed increased. This trend is a natural result of the reduced residence time of the hot air over a single area of the base material. As shown in Figure 8b, the mean deviation of preheating temperature tended to decrease as the lateral distance from the center of the hot-air stream increased. Thermocouples located at positions 1–3, which directly received the hot-air stream, exhibited relatively larger mean deviations because they were more affected by the residence time of the hot-air on the HDPE base material. In contrast, thermocouples at positions 5–6, which were not directly exposed to the hot-air stream, were less affected by hot-air movement speed because their temperature increase primarily resulted from heat conduction within the HDPE material.

## 4. Analysis and Discussion

### 4.1. Tensile Strength Results

The tensile strength of welded high-density polyethylene (HDPE) represents the maximum stress that the welded joint can withstand under uniaxial tension before failure. It serves as a critical indicator of weld quality, reflecting the integrity of the joint, including the presence of voids, bead uniformity, and overall fusion. Higher tensile strength corresponds to superior mechanical performance and durability of the welded component. In the context of medical device design and manufacturing, the tensile strength of HDPE welds plays a pivotal role in both the design and fabrication phases. During design, tensile strength ensures that welded joints can withstand expected service loads while meeting required safety factors. During manufacturing, tensile testing provides a quantitative basis for quality control, enabling verification of welding parameters such as preheating, extrusion rate, and travel speed. Consequently, sufficient tensile strength is essential to prevent structural failure, leakage, and long-term reliability issues in HDPE-based medical device components and fluid delivery systems. In this study, the relationship between preheating and the tensile strength of HDPE welds is specifically investigated to elucidate the effects of thermal preparation on weld performance.

**Figure 9:**
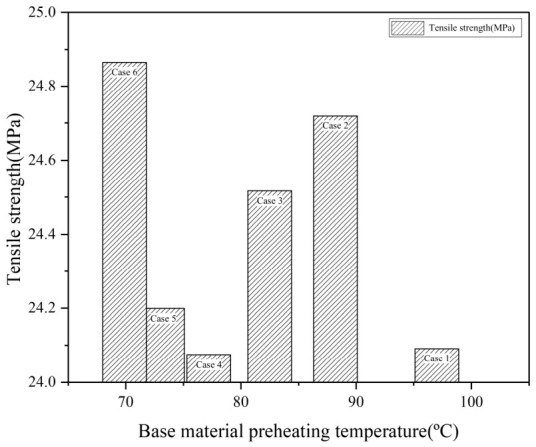
The tensile strength according to the preheating temperature.

**Figure 10:**
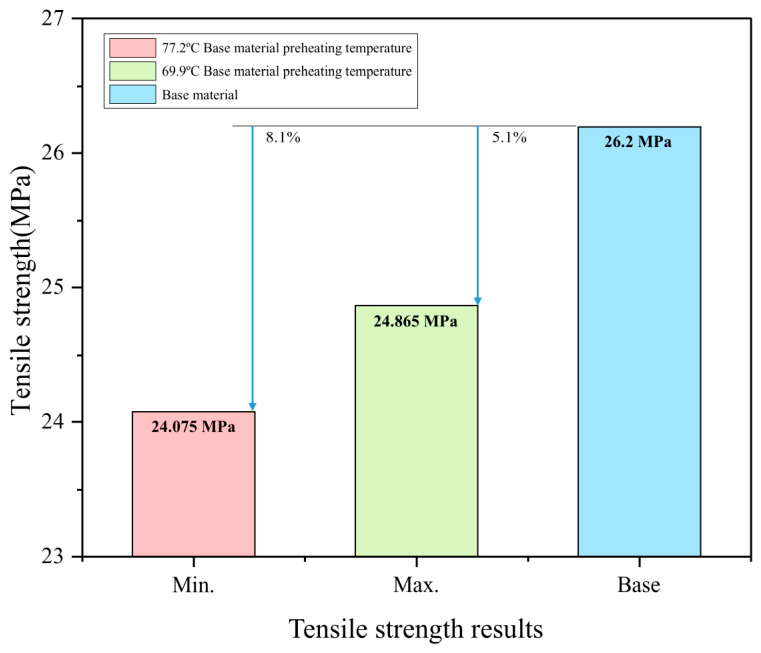
Comparison of tensile strength with the base material.

Figure 9 shows the relationship between the maximum preheating temperature and the tensile strength of the welded joint on the base material surface. Figure 10 compares the tensile strength of the welds with that of the base material. As shown in Figure 9 and Figure 10, no clear trend was observed between tensile strength and either the hot-air movement speed or the preheating temperature of the base material surface. Furthermore, as indicated in Figure 10, the difference in tensile strength with respect to the preheating temperature was relatively small. The difference between the maximum and minimum tensile strength values was 0.79 MPa, corresponding to less than 3%, which makes it difficult to conclude that variations in preheating temperature caused by hot-air movement speed have a decisive effect on weldability.

Although the tensile strength of the welded specimens was slightly lower than that of the base material, the reduction was less than 10%. In general, for HDPE materials, a weld joint is considered acceptable if the tensile strength reaches at least 80% of that of the base material. Therefore, all six welds produced under varying hot-air movement speed can be regarded as having formed good joints.

### 4.2. Bending Strength Results

The bending test was performed to evaluate the flexural performance of the welded HDPE specimens. This test applies a bending moment to the specimen, allowing measurement of key parameters such as flexural strength, flexural modulus, and strain at fracture. The results provide insight into the mechanical integrity and quality of the weld, complementing tensile strength measurements. In particular, the bending test enables assessment of joint uniformity, crack initiation, and failure modes, which are critical for predicting the performance of HDPE welds under actual structural bending conditions.

**Figure 11:**
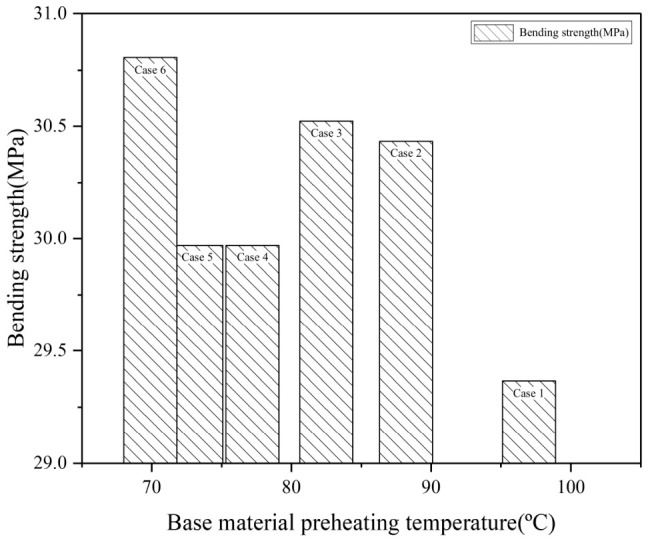
The bending strength according to preheating temperature.

Figure 11 shows the bending strength according to the preheating temperature at the surface of the base material. Similar to the tensile strength results in Figure 9, no clear trend was observed in Figure 11 between bending strength and either the hot-air movement speed or the preheating temperature of the base material surface. However, as with the tensile strength results, it was found that the specimens with a base material surface preheating temperature of 69.9 °C exhibited slightly higher tensile and bending strengths compared to those welded at a preheating temperature of 97 °C.

**Figure 12:**
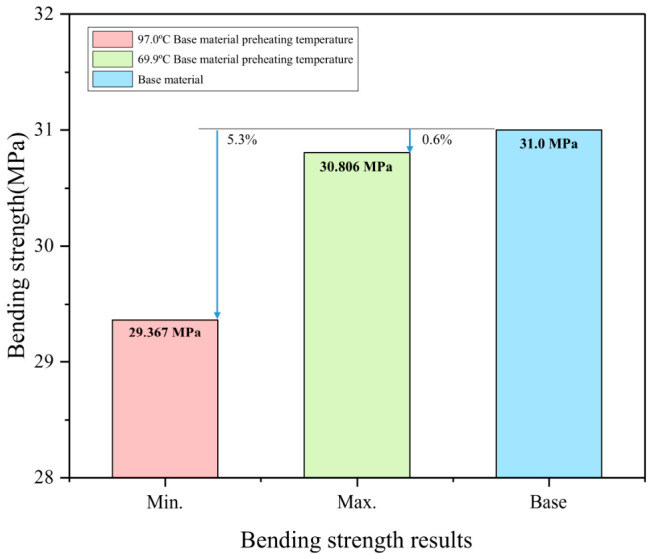
Comparison of bending strength with base material.

Figure 12 compares the bending strength of the welds with that of the base material. The difference between the max and min bending strength values with respect to the preheating temperature was 1.439 MPa, corresponding to an insignificant variation of 5%. Similar to the tensile test results, the bending strength data do not provide sufficient evidence to conclude that differences in preheating temperature caused by hot-air movement speed have a significant effect on weldability. The bending strength of the welds was reduced by 0.6–5.3% compared to that of the base material.

### 4.3. Discussion

Based on a previous study [19], it was assumed that the preheating condition of the base material would have a significant influence on the welding performance of HDPE, and this study was conducted to determine the appropriate preheating temperature conditions.

**Figure 13:**
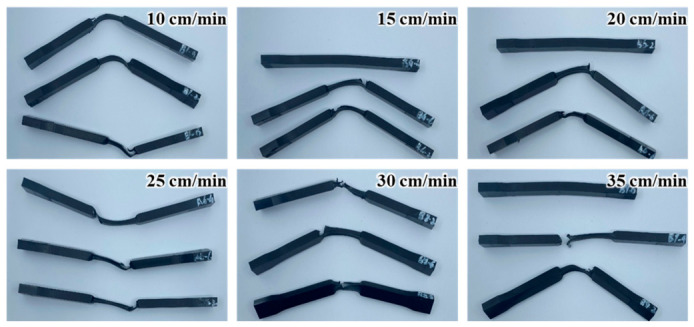
Fracture locations observed in tensile test specimens, illustrating the failure modes and positions relative to the welded region.

However, as shown in the tensile strength results (Section 4.1) and bending strength results (Section 4.2), no clear trend or significant correlation was observed with respect to the preheating temperature. This outcome can be inferred from the results presented in Figure 13, which show the fracture locations of the tensile specimens under different welding conditions. Tensile tests were conducted on specimens produced under six different conditions with varying extrusion discharge and hot-air movement speed, which resulted in different preheating temperatures at the base material surface.

In all six cases, a fracture occurred in the weld region rather than in the base material, indicating that the weld region has lower strength compared to the base material. Furthermore, as seen in Figure 9, the comparison of tensile strength between the base material and the welds shows that the difference between the maximum and minimum tensile strengths is 0.79 MPa, corresponding to less than 3%, and even the lowest tensile strength exhibited only an 8.1% reduction compared to the base material. These results confirm that good welds were achieved under all conditions.

**Figure 14:**
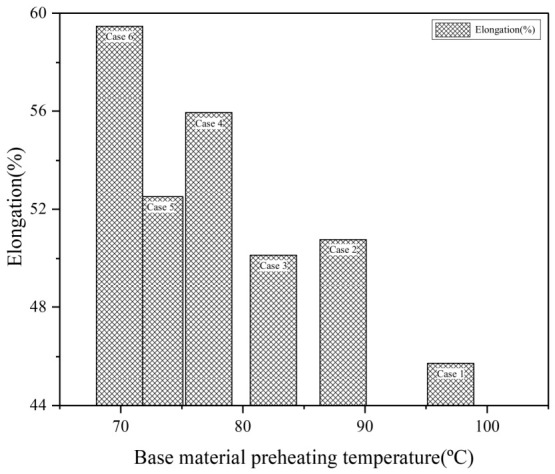
Variation in elongation of HDPE welds under different preheating temperatures, illustrating the effect of thermal preparation on ductility.

**Figure 15:**
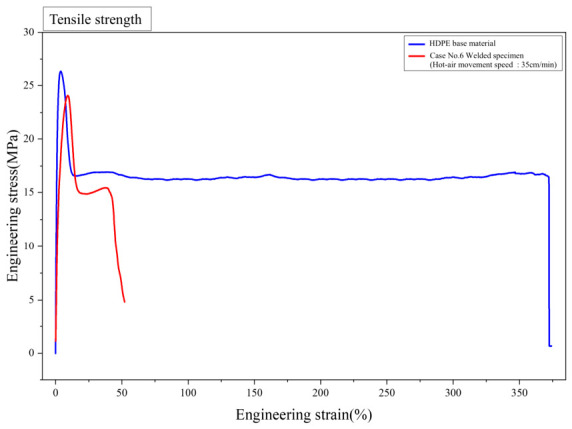
Tensile stress–strain curves of the base material and the welded specimen.

Figure 14 shows the elongation results of the welded joints. Figure 15 shows the tensile stress–strain curves of the base material and the welded specimen. Unlike the tensile and bending strength results discussed earlier, the elongation exhibited a relative trend. As the preheating temperature at the base material surface increased, the elongation tended to decrease. The welded joints show considerably lower elongation values compared to the base material, which had elongation above 300%.

This behavior is attributed to the relaxation of polymer chains during the preheating process. Because the polymer chains have varying lengths, each chain relaxes at a different temperature; however, as the preheating temperature increases, a greater number of polymer chains undergo relaxation. Polymer relaxation leads to the observed reduction in mechanical properties [35,36].

Therefore, depending on the preheating conditions, the maximum tensile and bending strengths of the HDPE welds exhibited similar values, while the elongation showed a distinct variation with increasing preheating temperature. This indicates that preheating primarily affects the elongation behavior, whereas its influence on tensile and bending strength is relatively minor. The similar tensile and bending properties can be attributed to comparable crystallization behavior during cooling, as all welds were air-cooled under identical conditions [37]. Because the mechanical properties of semi-crystalline polymers are largely determined by their degree of crystallinity, once sufficient preheating is achieved to enable proper fusion, the cooling process becomes the dominant factor governing the final strength. In contrast, elongation is more sensitive to the surface temperature of the base material during welding, where polymer chain alignment and partial crystallization occur at the interface. Consequently, it can be concluded that elongation is determined during the welding stage, while tensile and bending strengths are primarily established during cooling. Within the experimental preheating range, all welds exhibited comparable maximum strength, indicating that this temperature range is suitable for HDPE extrusion welding. Considering both elongation behavior and weldability, preheating conditions corresponding to hot-air movement speeds of 25–35 cm/min are recommended as optimal.

## 5. Conclusions

In this study, the effect of preheating the base material surface on the mechanical properties of HDPE joints was experimentally investigated. Based on the influence of the base material’s surface preheating temperature on the weld properties, the following conclusions were drawn from the analysis of the experimental results:

1. The surface temperature distribution during preheating depended primarily on the lateral distance from the heat source rather than the vertical position, confirming that heat conduction within HDPE governs thermal uniformity during hot-air preheating.
2. Increasing the hot-air movement speed reduced the surface preheating temperature linearly due to shortened exposure time. However, these temperature variations produced less than a 5% change in tensile and bending strengths, indicating that HDPE joints remain structurally reliable across a wide preheating range.
3. All weld specimens fractured within the welded region but still achieved tensile strengths exceeding 90% of the base material, satisfying the acceptance criteria defined in DVS standards.
4. Elongation decreased with increasing preheating temperature, likely due to polymer chain relaxation or localized degradation at elevated temperatures. Nevertheless, an optimal trade-off between strength and ductility was achieved at a surface temperature of approximately 70 °C with a hot-air speed of 35 cm/min.
5. These findings demonstrate that excessive preheating offers limited mechanical benefit, whereas moderate preheating ensures consistent joint strength, stable ductility, and reduced thermal distortion. The results provide practical guidance for selecting energy-efficient and reproducible process parameters in HDPE extrusion welding for medical electrical equipment applications.

## Figures and Tables

**Table 1:** Physical and thermal properties of the HDPE base material and filler wire used in the extrusion welding experiments.

Properties	Base Material	Filler Wire
Density (g/cm^3^)	0.95	0.945
Elongation (%)	≤500	-
Tensile strength (MPa)	26.2	-
Bending strength (MPa)	31	-
Melt index (g/10 min), 190 °C/5 kg	0.22	0.27
Melting point (°C)	120	120

**Table 2:** HDPE welding conditions.

Case No.	Hot-Air Moving Speed (cm/min)	Material Throughput (g/s)	Wire Melting Temperature (°C)	Hot-Air Temperature (°C)	Pressure (kgf)
1	10	0.2	240	260	10
2	15	0.3	240	260	10
3	20	0.4	240	260	10
4	25	0.5	240	260	10
5	30	0.6	240	260	10
6	35	0.7	240	260	10

**Table 3:** Measured bead geometry of welded specimens under different extrusion rates and travel speeds.

Case No.	Hot-Air Moving Speed (cm/min)	Material Throughput (g/s)	Bead Width (mm)	Bead Height (mm)
1	10	0.2	25.50	3.48
2	15	0.3	25.15	2.62
3	20	0.4	25.89	3.22
4	25	0.5	25.48	3.37
5	30	0.6	24.10	3.05
6	35	0.7	25.05	2.45

## Data Availability

The original contributions presented in this study are included in the article. Further inquiries can be directed to the corresponding authors.

## References

[1] Vidakis N., Petousis M., Michailidis N., Mountakis N., Papadakis V., Argyros A., Charou C. (2023). Polyethylene glycol and polyvinylpyrrolidone reduction agents for medical grade polyamide 12/silver nanocomposites development for material extrusion 3D printing: Rheological, thermomechanical, and biocidal performance. React. Funct. Polym..

[2] Chauhan P., Sharma A.K., Lamba B.Y. (2026). Transforming medical plastic waste into valuable hydrocarbon fuels: Advances, challenges, and future opportunities. J. Anal. Appl. Pyrolysis.

[3] Mejia E., Cherupurakal N., Mourad A.-H.I., Al Hassanieh S., Rabia M. (2021). Effect of Processing Techniques on the Microstructure and Mechanical Performance of High-Density Polyethylene. Polymers.

[4] Gopanna A., Rajan K.P., Thomas S.P., Chavali M. (2019). Polyethylene and Polypropylene Matrix Composites for Biomedical Applications. Mater. Biomed. Eng..

[5] Niu S., Bellala V., Qureshi D.A., Srivastava V. (2024). A Method to Measure the Embedded Crack Length and Position in High-Density Polyethylene Using Ultrasound. arXiv.

[6] Luo H., Liu X., Chen X., Pu Z., Jia Z., Yue J. (2026). Engineering surfaces of polymer-based medical implants for tissue repair and regeneration. J. Control. Release.

[7] Langer R., Tirrell D.A. (2004). Designing materials for biology and medicine. Nature.

[8] Jones D.S., McCoy C.P., Andrews G.P. (2011). Physicochemical and drug diffusion analysis of rifampicin containing polyethylene glycol–poly(ɛ-caprolactone) networks designed for medical device applications. Chem. Eng. J..

[9] Maro M.D., Faga M.G., Malucelli G., Mussano F.D., Genova T., Morsi R.E., Hamdy A., Duraccio D. (2020). Influence of Chitosan on the Mechanical and Biological Properties of HDPE for Biomedical Applications. Polym. Test..

[10] Paxton N.C., Allenby M.C., Lewis P.M., Woodruff M.A. (2019). Biomedical applications of polyethylene. Eur. Polym. J..

[11] Amjadi M., Fatemi A. (2020). Tensile Behavior of High-Density Polyethylene Including the Effects of Processing Technique, Thickness, Temperature, and Strain Rate. Polymers.

[12] Al-mugren K.S., Almalki L., Alshehri R., Alamri S., Almurayshid M., Alsuhybani M., Alharbi R., Khandaker M.U. (2024). Development of lead-free metal carbides and ceramic decorated HDPE composites for low energy X-ray shielding applications. Radiat. Phys. Chem..

[13] Gebrehiwot S.Z., Espinosa-Leal L., Anukka H., Remes H. (2025). Modelling the Effect of Temperature on the Plastic Deformation of High-Density Polyethylene (HDPE): A Semi-Empirical Approach. Mech. Mater..

[14] Mehdikhani H., Mostafapour A., Laieghi H., Najjar R., Lionetto F. (2022). Mechanical and Microstructural Properties of HDPE Pipes Manufactured via Orbital Friction Stir Welding. Materials.

[15] Ageorges C., Ye L., Hou M. (2001). Advances in Fusion Bonding Techniques for Joining Thermoplastic Matrix Composites: A Review. Compos. Part A Appl. Sci. Manuf..

[16] Grimm R.A. (1995). Welding processes for plastics. Adv. Mater. Process..

[17] Huang Y., Meng X., Xie Y., Wan L., Lv Z., Cao J., Feng J. (2018). Friction stir welding/processing of polymers and polymer matrix composites. Compos. Part A Appl. Sci. Manuf..

[18] Schmachtenberg E., Tüchert C. (2003). Long-term properties of butt-welded poly(propylene). Macromol. Mater. Eng..

[19] Lee C., Woo S., Kwon S., Kim J. (2024). Effect of Preheating Parameters on Extrusion Welding of High-Density Polyethylene Materials. Polymers.

[20] Amanat N., James N.L., McKenzie D.R. (2010). Welding Methods for Joining Thermoplastic Polymers for the Hermetic Enclosure of Medical Devices. Med. Eng. Phys..

[21] Rowe R.K., Ali M.M. (2024). Effect of Welding Parameters on Properties of HDPE Geomembrane Extrusion Welds. Geotext. Geomembr..

[22] Lai H., Fan D., Liu K. (2022). The Effect of Welding Defects on the Long-Term Performance of HDPE Pipes. Polymers.

[23] Ting G., Grest G.S., Robbins M.O. (2014). Tensile Fracture of Welded Polymer Interfaces: Miscibility, Entanglements, and Crazing. Macromolecules.

[24] Wang S., Shi J., Shimizu T., Xu J., Xu Z. (2022). Two-Step Heat Fusion Kinetics and Mechanical Performance of Thermoplastic Interfaces. Sci. Rep..

[25] Bai C., Lin R., Lai H.S. (2024). Investigation of Creep Behavior of HDPE Pipe Butt Fusion Welded Joints Using a Stepped Isostress Method. Polymers.

[26] Ge T., Pierce F., Perahia D., Grest G.S., Robbins M.O. (2013). Molecular Dynamics Simulations of Polymer Welding: Strength from Interfacial Entanglements. Phys. Rev. Lett..

[27] Pokharel P., Kim Y., Choi S. (2016). Microstructure and Mechanical Properties of the Butt Joint in High Density Polyethylene Pipe. Int. J. Polym. Sci..

[28] Cai Z., Dai H., Fu X. (2018). Investigation on the Hot Melting Temperature Field Simulation of HDPE Water Supply Pipeline in Gymnasium Pool. Results Phys..

[29] Dai H., Peng J. (2017). The Effects of Welded Joint Characteristics on Its Properties in HDPE Thermal Fusion Welding. Mod. Phys. Lett. B.

[30] Luchinsky D.G., Hafiychuk H., Hafiychuk V., Chaki K., Nitta H., Ozawa T., Wheeler K.R., Prater T.J., McClintock P.V.E. (2020). Welding Dynamics in an Atomistic Model of an Amorphous Polymer Blend with Polymer–Polymer Interface. J. Polym. Sci. Part B Polym. Phys..

[31] Schnell R., Stamm M., Creton C. (1998). Direct Correlation between Interfacial Width and Adhesion in Glassy Polymers. Macromolecules.

[32] Riahi M., Kooshayan K., Ghanati M.F. (2011). Analysis of effect of pressure and heat on mechanical characteristics of butt fusion welding of polyethylene pipes. Polym. Plast. Technol. Eng..

[33] Ezekoye O.A., Lowman C.D., Fahey M.T., Hulme-Lowe A.G. (1998). Polymer Weld Strength Predictions Using a Thermal and Polymer Chain Diffusion Analysis. Polym. Eng. Sci..

[34] Rehman R.U., Sheikh-Ahmad J., Deveci S. (2021). Effect of Preheating on Joint Quality in the Friction Stir Welding of Bimodal High Density Polyethylene. Int. J. Adv. Manuf. Technol..

[35] Djoković V. (1999). Stress relaxation in high-density polyethylene: Effects of orientation and irradiation. Polym. J..

[36] Lomovskoy V.A. (2024). Relaxation phenomena in low-density and high-density polyethylenes. Polymers.

[37] Gao Y., Jayswal A., Das A., Carrillo J.M.Y., Damron J.T., Bowland C.C., Yu Z., Toomey M., Angelopoulou P.P., Kharal S. (2025). Enhanced interfacial bonding of graft copolymers. ACS Appl. Mater. Interfaces.

